# The association between the volume of the gallbladder based on sonographic findings and demographical data in the PERSIAN Guilan cohort study (PGCS)

**DOI:** 10.1186/s13104-023-06582-x

**Published:** 2023-11-03

**Authors:** Farahnaz Joukar, Mohammad Taghi Ashoobi, Ahmad Alizadeh, Tahereh Zeinali, Niloofar Faraji, Mohammadjavad Tabatabaii, Roya Mansour-Ghanaei, Mohammadreza Naghipour, Fariborz Mansour-Ghanaei

**Affiliations:** 1 Gastrointestinal and Liver Diseases Research Center, Guilan University of Medical Sciences, Rasht, Iran; 2 Department of Surgery, School of Medicine Razi Hospital, Guilan University of Medical Sciences, Rasht, Iran; 3 Department of Radiology, School of Medicine, Poursina Hospital, Guilan University of Medical Sciences, Rasht, Iran

**Keywords:** Gallbladder, Sonography, Fasting gallbladder volume

## Abstract

**Background:**

Ultrasound is an important method to determine the volume of the gallbladder and check its structure. Considering the variation in the size and volume of the gallbladder in disease and physiological conditions, determining the volume of the gallbladder is clinically valuable. This study was carried out to evaluate the gallbladder volume and its association with patients’ demographic data in the Prospective Epidemiological Research Studies of Iranian Adults (PERSIAN) Guilan cohort study (PGCS) population.

**Methods:**

In this cross-sectional study, 957 individuals aged 35–70 participated in determining the gallbladder volume by a radiologist based on the ultrasound method. The demographical data were collected using a questionnaire. After fasting for 12 h, the ultrasound was performed with an Ultrasonic device (Sonix SP series) with a 3.5 to 5 MHz probe.

**Results:**

The total frequency of gallbladder lesions was 2.2%. The results showed a significant association between marriage and gender with the presence or absence of lesions in the studied participants (*P* < 0.05). Also, significant differences were reported between the volume of gallbladder and gender, body mass index (BMI), social and economic status (SES), metabolic equivalent of task (MET), history of cardiovascular disease (CVD), and hypertension (*P* < 0.05). The results of a linear regression represented a significant association between gender, BMI, MET, and CVD and the mean volume of the gallbladder (*P* < 0.05). However, there was no significant association between the presence or absence of a lesion and the individuals’ average gallbladder volume (*P* > 0.05).

**Conclusion:**

According to our results, gender, BMI, MET, and CVD were significantly associated with gallbladder volume.

## Introduction

Gallbladder volume (GBV) can reflect clinical and therapeutic implications, physiological and functional status, and possibly pathophysiological mechanisms of gallstone diseases. Gallbladder lesions, such as stones and sludge, are the most common biliary system complications that lead to the hospitalization of patients [1, 2]. Much attention has been paid to related risk factors and epidemiological factors in the last two decades. However, today, the prevalence and epidemiology of this disease are changing due to changes in lifestyles and the industrialization of societies [3]. This complication imposes a significant medical-economic burden on the health system. Even in Western countries, 10% of people in European and American societies have gallstones [4, 5].

Gallbladder lesions are common in adults, especially middle-aged women [6, 7]. Although ultrasound is very accurate in diagnosis, better diagnosis is provided by magnetic resonance cholangiopancreatography (MRCP) [8–10]. Gallbladder sludges and stones usually cause symptoms due to inflammation or obstruction caused by their entry into the cystic or common bile duct (CBD) [11]. Gallstones with a naturally “silent” or asymptomatic nature have been more controversial among gallbladder lesions. It has been observed that patients who remain asymptomatic for 15 years are unlikely to develop symptoms during follow-up, and most of the patients who suffered complications from gallstones had previous warning symptoms. There are similar results in diabetic patients with silent gallstones [12].

Since epidemiological studies have not been conducted to investigate the relationship between gallbladder volume and risk factors in this region, and considering the importance of gallbladder volume, which can be a predictor of increasing the risk of gallbladder complications, we conducted this study to determine the association of the volume of the gallbladder based on ultrasound findings and demographical and clinical data of individuals in the Prospective Epidemiological Research Studies of Iranian Adults (PERSIAN) Guilan cohort study (PGCS) population.

## Methods and patients

### Participants

This cross-sectional study was conducted on 957 individuals selected from 10,520 individuals of the PGCS population aged 35–70 years through a sequential sampling method from 2014 to 2017 in Iran [13]. Written Consent was taken after informing each participant of the purpose and importance of the study to each participant. To ensure the confidentiality of the participant’s information, codes were used whereby the participant’s name and any participant identifier were not written on the questionnaire. This study was approved by the ethics committees at the Ministry of Health and Medical Education, the Digestive Diseases Research Institute (Tehran University of Medical Sciences), and Guilan University of Medical Sciences. Informed Consent was obtained from all individual participants.

### Data collecting

The PERSIAN Cohort questionnaire (validated for our population) in a face-to-face interview format and a physical examination were accomplished. In the general sector, an eleven-digit code was allocated to each participant. In this phase, the following data were collected (demographic data and clinical characteristics). Anthropometric indices, including weight (in kg), height, hip, waist, and wrist circumference (in cm), were measured according to the National Health and Nutrition Examination Survey Manual. Participants were classified into the following body mass index (BMI) groups: underweight (BMI < 18.5 kg/m2), average weight (BMI = 18.5–24.99 kg/m2), overweight (BMI = 25–29.9 kg/m2) and obese (BMI ≥ 30 kg/m2). Physical activity according to the metabolic equivalent of task (MET) was divided into three tertiles by low (< 36.1), medium (36.1–42.8), and high (> 42.8) levels of activity in a day by measuring the number of hours of walking, working, exercise, etc.; and social and economic status (SES) was classified in three levels low, middle, and high level. A history of smoking and alcohol consumption also recorded. Hypertension was definited as a systolic blood pressure ≥ 140 mm Hg or a diastolic blood pressure ≥ 90 mm Hg, or a previous physician diagnosis of hypertension, or use of antihypertensive medications. CVDs comprised to history of cardiac ischemia and/or myocardial infarction and/or stroke.

The criteria for diabetes were self-reporting, taking glucose-lowering medication, and the result of fasting blood sugar equal to or higher than 126 mg/dL [13, 14]. Not having chronic and acute liver disease including viral hepatitis C, B, chronic or acute kidney disease, cancers, pregnancy, taking medications affecting the liver such as steroids, amiodarone, tamoxifen, biliary diseases, and patients with proven hemochromatosis, were considered as the exclusion criteria for this study, and identification of the subjects was conducted based on the file created in the cohort plan. A radiologist performed ultrasonography after at least 12 h of fasting, using an Ultrasonic Device of Sonix SP type with 3.5 MHz to 5 MHz probes to determine the gallbladder volume.

### Statistical analysis

Continuous variables were expressed as mean and standard deviation (SD), and the categorical variables was reported frequency and percentages. The normality was evaluated using the Shapiro-Wilk normality test. Absolute and relative frequency, mean, and standard deviation were used to describe the data, and the Chi-Square test was used to determine the relationship between variables. Linear regression was applied to evaluate the association between variables. Statistical analysis was undertaken using SPSS software (version 16.0) at a significance level 0.05.

## Results

The gallbladder lesions were examined by ultrasound in 957 individuals, of whom 37.7% (n = 361) were female. About 83.2% (n = 797) of individuals aged 35–55, and 16.7% (n = 160) were over 55. The majority of participants were married 95.3% (n = 912), at a diploma level of education 56.7% (n = 543), with low and high levels of SES and MET, 367 (38.3%) and 372 (38.8%) respectively, with no history of smoking 69.3% (n = 663) or alcohol consumption 95.6% (n = 915), and non-diabetic 90.3% (n-864), Table 1. The total frequency of gallbladder lesions was 2.2% (n = 21). The females had a higher frequency of gallbladder lesions, 3.3% (n = 12), than males, 1.5% (n = 9) among patients with gallbladder lesions. The high frequency of gallbladder lesions in married people was 2.1% (n = 19), and in widowed people, it was 2% (n = 20).

**Table 1:** Comparing the presence of lesions and demographic data of the PERSIAN Guilan cohort study population

Demographical data		Total n = 957 (%)	Gallbladder lesion n (%)		*P value*
			Yes 21 (2.2)	No 936 (97.8)	
**Age (year)**	*35–45*	435 (45.4)	7 (1.6)	428 (98.4)	0.300
	*45–55*	362 (37.8)	12 (3.3)	350 (96.7)	
	*> 55*	160 (16.7)	2 (1.3)	158 (98.8)	
**Gender**	*Female*	361 (37.7)	12 (3.3)	349 (96.7)	0.050
	*Male*	596 (62.3)	9 (1.5)	587 (98.5)	
**Marital status**	*Single*	29 (3)	0 (0)	29 (100)	0.001
	*Married*	912 (95.3)	19 (2.1)	893 (97.9)	
	*Divorce*	6 (0.6)	0 (0)	6 (100)	
	*Widow*	10 (1)	2 (20)	8 (80)	
**Educational level**	*Illiterate*	64 (6.4)	2(3.1)	62 (96.8)	0.600
	*Primary school*	207 (21.6)	3 (1.4)	204 (98.6)	
	*Diploma*	543 (56.7)	12 (2.2)	531 (97.8)	
	*University degree*	143 (15.3)	4 (2.7)	139 (97.2)	
**Occupation**	*Farmer*	109 (11.4)	2 (1.8)	107 (98.2)	0.090
	*Employee*	164 (17.1)	2 (1.2)	162 (98.8)	
	*Simple worker*	133 (13.9)	1 (0.8)	132 (99.2)	
	*Student*	6 (0.6)	1 (16.7)	5 (83.2)	
	*Housewife*	308 (32.2)	10 (3.2)	298 (96.8)	
	*Self-employee*	237 (24.8)	5 (2.1)	232 (97.9)	
**BMI**	*< 25*	237 (24.8)	6 (2.5)	231 (97.5)	0.800
	*25–30*	432 (45.1)	8 (1.9)	424 (98.1)	
	*> 30*	288 (30.1)	7 (2.4)	281 (97.6)	
**SES**	*Low*	367 (38.3)	7 (1.9)	360 (98.1)	0.725
	*Moderate*	347 (36.2)	7 (2.0)	340 (98.0)	
	*High*	243 (25.3)	7 (2.9)	236 (97.1)	
**MET**	*Low*	244 (25.4)	3 (1.2)	241 (98.8)	0.360
	*Moderate*	341(35.6)	7 (2.1)	334 (97.9)	
	*High*	372 (38.8)	11 (3.0)	361 (97.0)	
**Smoking**	*No*	663 (69.3)	16 (2.4)	647 (97.6)	0.300
	*Yes*	294 (30.7)	5 (1.7)	289 (98.3)	
**Alcohol consumption**	*No*	915 (95.6)	20 (2.2)	895 (98.8)	0.600
	*Yes*	42 (4.4)	1 (2.4)	41 (97.6)	
**Diabetes**	*No*	864 (90.3)	19 (2.2)	845 (97.8)	0.600
	*Yes*	93 (9.7)	2 (2.2)	531 (97.8)	
**Hypertension**	*No*	682 (71.2)	17 (2.5)	665 (97.5)	0.349
	*Yes*	275 (28.8)	4 (1.5)	271 (98.5)	
**History of CVD**	*No*	857 (89.5)	19 (2.2)	857 (97.8)	0.696
	*Yes*	79 (10.5)	2 (2.5)	79 (97.5)	

Body mass index (BMI), Social and economic status (SES), Metabolic equivalent of task (MET), Cardiovascular disease (CVD).

The mean size of the gallbladder volume is 4 ± 32 cm^3^. About 58% (n = 559) of participants represented a gallbladder volume lower than average size. Based on the analysis, there was a significant association between the average volume of the gallbladder and gender, marital status, BMI, MET, history of smoking, alcohol consumption, hypertension, and CVD among participants (*P* < 0.05), Table 2. There is no association between the gallbladder’s mean volume and the gallbladder lesion’s presence (*P* > 0.05). The results of the linear regression reported a significant association between gender, MET, BMI, and history of CVD with the volume of the gallbladder by elimination of the confounders (*P* < 0.05) (Table 3).

**Table 2:** Comparison the mean of gallbladder volume and demographic data of the PERSIAN Guilan cohort study population

Demographical data		Gallbladder volume Mean ± SD	*P value*
**Age (year)**	*35–45*	32.37 ± 12.33	0.200
	*45–55*	31.84 ± 11.62	
	*> 55*	33.81 ± 16.16	
**Gender**	*Female*	29.33 ± 10.31	0.001
	*Male*	34.32 ± 13.81	
**Marital status**	*Single*	26.07 ± 9.47	0.020
	*Married*	32.07 ± 12.92	
	*Divorce*	28.57 ± 9.94	
	*Widow*	27.20 ± 7.06	
**Educational level**	*Illiterate*	31.27 ± 9.01	0.700
	*Primary school*	31.89 ± 13.96	
	*Diploma*	32.78 ± 13.30	
	*University degree*	31.09 ± 11.35	
**Occupation**	*Farmer*	33.30 ± 12.51	0.500
	*Employee*	33.34 ± 11.77	
	*Simple worker*	32.12 ± 10.83	
	*Student*	33.30 ± 12.51	
	*Housewife*	31.23 ± 9.70	
	*Self-employee*	32.11 ± 12.88	
**BMI**	*< 25*	29.75 ± 12.15	0.004
	*25–30*	29.75 ± 12.95	
	*> 30*	34.45 ± 14.21	
**SES**	*Low*	33.40 ± 14.74	0.143
	*Moderate*	32.10 ± 11.48	
	*High*	31.41 ± 11.46	
**MET**	*Low*	34.84 ± 15.88	0.002
	*Moderate*	32.03 ± 12.82	
	*High*	31.20 ± 10.10	
**Smoking**	*No*	35.46 ± 15.30	0.001
	*Yes*	31.82 ± 12.19	
**Alcohol consumption**	*No*	32.07 ± 12.86	0.007
	*Yes*	35.96 ± 11.87	
**Diabetes**	*No*	32.16 ± 12.48	0.060
	*Yes*	34.68 ± 15.40	
**Hypertension**	*No*	31.83 ± 11.01	0.024
	*Yes*	33.90 ± 16.40	
**History of CVD**	*No*	32.01 ± 11.58	0.001
	*Yes*	36.94 ± 21.74	

Body mass index (BMI), Social and economic status (SES), Metabolic equivalent of task (MET), Cardiovascular disease (CVD).

**Table 3:** Multiple Linear regression analysis for association between demographic and clinical factors among patients according to the volume of gallbladder among the PERSIAN Guilan cohort study population

Variables	β	Standard error	Lower limit	Upper limit	*P* value
**Age**	0.04	0.05	− 0.07	0.15	0.461
**Gender**	5.98	1.01	4.01	7.94	< 0.001
**Education**	− 0.38	0.39	-1.14	0.38	0.325
**BMI**	0.61	0.09	0.42	0.78	< 0.001
**SES**	− 0.56	0.50	-1.55	0.42	0.260
**MET**	-1.21	0.50	-2.20	-0.22	0.017
**Smoking**	-1.42	1.01	-3.39	0.53	0.154
**Alcohol consumption**	0.08	0.51	-1.08	0.92	0.870
**Diabetes**	2.46	1.36	-0.21	5.14	0.072
**Hypertension**	1.09	0.92	-0.71	2.89	0.236
**History of CVD**	4.15	1.49	1.22	7.09	0.006

Body mass index (BMI), Social and economic status (SES), Metabolic equivalent of task (MET), Cardiovascular disease (CVD).

## Discussion

Gallbladder lesions are the most common disorders in the biliary system that cause many economic and health problems in most countries. In the last two decades, much attention has been paid to the risk factors and epidemiological factors of gallbladder lesions [15–17]. The mean gallbladder volume in this study (32 ± 4 cm^3^) is comparable with the findings of Ikhuoriah et al. (34.51 + 3.16cm^3^) in their comparative study on 120 patients with type 2 diabetes and 120 non-diabetic controls [18]. Akintomide et al. reported a lower mean gallbladder volume of 27.7 ± 12.3 cm^3^ of 141 participants in southwest Nigeria [4]. The correlation of gallbladder volume from this study with those with larger sample sizes suggests that gallbladder volume shows no significant variation between ethnic groups, countries, and possible races [19]. Huang et al. indicated the gallbladder volume was more significant in the late stage than in early/second stage acute cholecystitis gallbladders (84.66 ± 26.32 cm^3^, vs. 53.19 ± 33.80 cm^3^). The fasting volume/ejection fraction of gallbladders in chronic cholecystitis was more significant/lower than those of normal subjects [20].

In the present study, it has been determined that the gallbladder volume increased with increasing BMI, which is consistent with other studies. Idris et al. determined the gallbladder volume associated with age, gender, and BMI. The results showed a statistically significant difference between BMI and gallbladder volume [19]. Also, Caroli-Bosc et al. obtained similar results that a positive statistical difference was observed between the volume of the gallbladder and the BMI [21]. The etiology of gallbladder disease involves multiple mechanisms found in individuals with high BMI. These include excessive hepatic cholesterol secretion and subsequent bile saturation, gallbladder inflammation and fatty degeneration (cholecystosteatosis), and slowed gallbladder contraction [22].

Shanmugam et al. reported the association between physical activity and gallbladder diseases [23]. The current study demonstrated a significant association between MET and gallbladder volume. Physical activity benefits gallbladder function and helps reduce the risk of gallstones and associated symptoms and complications. The positive effects of physical activity on gallbladder function include promoting gallbladder motility, preventing stagnation of bile, aiding in weight management, improving insulin sensitivity, and enhancing metabolic health. These findings highlight the importance of incorporating regular physical activity to maintain gallbladder health and reduce the risk of gallstone-related issues.

In this study, marital status represented no statistically significant association with gallbladder volume. Few studies report a correlation between gallbladder disease and marital status [24, 25]. The study of Bilal et al. indicated that gallbladder disease was more common in single, widow/separated females than our results [26]. Since marital status influences patient diagnosis and treatment and married patients have been reported to have a better prognosis with early diagnosis and treatment [26, 27], there is a need for more studies to determine the relationship between marital status and gallbladder diseases.

One of the considerable demographical factors associated with greater gallbladder volume in the current study was the level of SES. Studies reported different results on the association between gallbladder complications and SES [28, 29]. SES can influence dietary patterns and lifestyle choices, impacting gallbladder health. Individuals with higher SES often have better access to healthier food options, engage in regular physical activity, and have lower rates of obesity. These factors, in turn, can contribute to better gallbladder health and potentially impact gallbladder volume. Socioeconomic disparities can affect access to healthcare services, including preventive care and timely treatment. Also, limited access to healthcare may result in delayed diagnosis and treatment of gallbladder-related conditions, such as gallstones. Delayed intervention can lead to complications that may influence gallbladder volume.

Our findings revealed that males have a higher mean gallbladder volume than females. A study on healthy adults in Kano, North-Western Nigeria, showed a positive correlation and variation with gender, in which the gallbladder volume was significantly higher in males than females [19]. Our study has established a significant correlation between gallbladder volume with gender and BMI, as indicated by the linear regression model, but no association was reported with age. In comparison, a recent study on the Iranian population indicated higher occurrence with the participants in the fourth decade of life or above [30]. However, As the gallbladder volume increases, Inah et al.‘s study showed a significant difference between age and gallbladder volume. This result might be reflected by various categorized into age groups [31].

In the current study, no association was reported between a history of smoking and alcohol consumption and gallbladder volume. On the contrary, Romański et al. showed that smoking as one of the independent risk factors leads to an increase in gallbladder volume, especially in men [32]. Kasicka-Jonderko et al. indicated that ethanol solutions caused inhibition and delay in gallbladder emptying. They reported that alcoholic beverages on an empty stomach inhibit gastrointestinal transport and gallbladder emptying [33].

In the present study, diabetes represented no statistically significant association with gallbladder volume. On the contrary, Ranganath et al. found that gallbladder volume in diabetic patients was significantly greater compared with that of controls [34]. While CVD and hypertension may not have a direct causal relationship with gallbladder volume, they can share common risk factors such as obesity, diabetes, and high cholesterol levels. These risk factors are controversially known to contribute to both CVD and hypertension and gallbladder-related issues such as gallstones [35–38]. In the current study, a history of CVD was associated with the mean volume of the gallbladder.

One of our limitations is the cross-sectional nature of this research.

## Conclusion

Due to our results, the gallbladder volume was significantly associated with gender, BMI, and the level of physical activity. Also, patients with CVD had a greater gallbladder volume.

## Data Availability

The corresponding author is available in the case requiring, and all data and tables, used in the manuscript are prepared originally by authors.
